# Growing in the city: Urban evolutionary ecology of avian growth rates

**DOI:** 10.1111/eva.13081

**Published:** 2020-09-17

**Authors:** Michela Corsini, Eva Maria Schöll, Irene Di Lecce, Marion Chatelain, Anna Dubiec, Marta Szulkin

**Affiliations:** ^1^ Centre of New Technologies University of Warsaw Warsaw Poland; ^2^ Institute of Wildlife Biology and Game Management University of Natural Resources and Life Sciences Vienna Austria; ^3^ Applied and Trophic Ecology Department of Zoology University of Innsbruck Innsbruck Austria; ^4^ Museum and Institute of Zoology Polish Academy of Sciences Warsaw Poland

**Keywords:** birds, body mass, growth rate, imperviousness, phenotypic divergence, selection, urbanization

## Abstract

**Introduction:**

Rapid environmental change driven by urbanization offers a unique insight into the adaptive potential of urban‐dwelling organisms. Urban‐driven phenotypic differentiation is increasingly often demonstrated, but the impact of urbanization (here modelled as the percentage of impervious surface (ISA) around each nestbox) on offspring developmental rates and subsequent survival remains poorly understood. Furthermore, the role of selection on urban‐driven phenotypic divergence was rarely investigated to date.

**Methods and Results:**

Data on nestling development and body mass were analysed in a gradient of urbanization set in Warsaw, Poland, in two passerine species: great tits (*Parus major*) and blue tits (*Cyanistes caeruleus*). Increasing levels of impervious surface area (ISA) delayed the age of fastest growth in blue tits. Nestling body mass was also negatively affected by increasing ISA 5 and 10 days after hatching in great tits, and 10 and 15 days in blue tits, respectively. High levels of ISA also increased nestling mortality 5 and 10 days after hatching in both species. An analysis of selection differentials performed for two levels of urbanization (low and high ISA) revealed a positive association between mass at day 2 and survival at fledging.

**Discussion:**

This study confirms the considerable negative impact of imperviousness—a proxy for urbanization level—on offspring development, body mass and survival, and highlights increased selection on avian mass at hatching in a high ISA environment.

## 1 INTRODUCTION

Urbanization leads to drastic and widespread alterations to both biotic and abiotic components of the environment (Grimm et al., 2008; McKinney, 2006). Currently, half of the world's human population lives in urbanized environments; this figure is predicted to increase to 66% over the next 20 years (United Nations, 2018). Concomitantly, urban areas are expanding worldwide. At present, cities and towns cover *c*. 3% of Earth's land, and this statistic is expected to increase considerably in the next decades (Seto, Fragkias, Güneralp, & Reilly, 2011; Seto, Güneralp, & Hutyra, 2012; United Nations, 2018). Urbanization usually involves habitat fragmentation and contrasted anthropogenic land use. Consequently, this leads to an alternation of high‐density human settlements, industrial areas and buildings that are often intermixed with managed green areas or with remnants of natural habitats (Grimm et al., 2008; McIntyre, Rango, Fagan, & Faeth, 2001). The environmental structuring of cities thus unarguably differs from natural habitats. Urbanization is also characterized by increased noise and light pollution levels (Dominoni, Quetting, & Partecke, 2013; Isaksson, 2010; Miller, 2006; Slabbekoorn & Ripmeester, 2008), and increased temperatures (the urban heat island effect Diamond & Martin, 2020; Marzluff, 2001). All these dimensions of the urban habitat covary with the proportion of Impervious Surface Area (ISA) in the environment (Szulkin, Garroway, Corsini, Kotarba, & Dominoni, 2020). Consequently, the urbanization process can be viewed as a valuable opportunity to study eco‐evolutionary changes in a heterogeneous landscape, and ISA is a valuable proxy that allows for straightforward comparisons of such effects for distinct locations within the city or between urban areas. Contrasted selective pressures may arise from such an urban mosaic, leading to possibly far‐reaching long‐term fitness consequences for urban‐dwelling organisms (Alberti, Marzluff, & Hunt, 2017; Johnson & Munshi‐South, 2017). Thus, the urbanization process can lead to both divergent phenotypes and rapid evolutionary change at rates exceeding those normally observed in nature (Alberti et al., 2017).

While many species cannot withstand the urban ecosystem structure and disappear once an area is urbanized (“urban avoiders”—Fischer, Schneider, Ahlers, & Miller, 2015; McKinney, 2002), others, known as “urban exploiters” and “urban adapters,” seemingly do well within urban environments. Although urban adapters take advantage of human‐provided resources similarly to urban exploiters, they do not strictly depend on them (Fischer et al., 2015; McKinney, 2006); thus, urban adapters provide an interesting opportunity to assess urban‐driven evolutionary change in free‐living populations. Importantly, even though urban adapters belong to a wide range of taxa (including: insects—Kaiser, Merckx, & Van Dyck, 2018, mammals—Harris, Munshi‐South, Obergfell, & O’Neill, 2013, amphibians—Stolyar, Loumbourdis, Falfushinska, & Romanchuk, 2008, reptiles—Winchell, Reynolds, Prado‐Irwin, Puente‐Rolón, & Revell, 2016; Winchell, Maayan, Fredette, & Revell, 2018), birds are an excellent model system to explore urbanization‐related shifts in evolutionary ecology (Chamberlain et al., 2009; Marzluff, 2017). Several studies emphasized important differences in the reproductive strategies adopted by rural and urban avian populations; thus, birds inhabiting cities often exhibit earlier laying dates, smaller clutches and reduced brood sizes compared to their rural counterparts (Chamberlain et al., 2009; de Satgé et al., 2019). Similarly, previous studies highlighted urbanization‐linked differences in adult body size and body condition, with urban birds being smaller and lighter relative to their rural conspecifics (Caizergues, Grégoire, & Charmantier, 2018; McDonnell & Hahs, 2015). Caizergues et al. (2018) reported phenotypic divergence between forest and urban populations, with urban adult great tits (*Parus major*) being smaller relative to their rural counterparts—a pattern also reported by Sprau, Mouchet, and Dingemanse (2017). Analogous differences were also measured in house sparrows (*Passer domesticus*), with urban‐dwelling individuals recorded as consistently smaller and with lower body conditions than individuals inhabiting rural habitats (Liker, Papp, Bókony, & Lendvai, 2008). Much less is known about the impact of urbanization on avian developmental patterns (Biard et al., 2017; Heiss, Clark, & McGowan, 2009; Salmon, Nilsson, Nord, Bensch, & Isaksson, 2016; Seress et al., 2012). Yet, nestling body mass at fledging—the time when hole‐nesting passerines leave the nest—is the end product of nestling development, and is well known to positively influence an individual's survival and recruitment to the breeding population (Nur, 1984; Tinbergen & Boerlijst, 1990). Thus, early developmental conditions in birds have important long‐term consequences for their future reproductive success (Metcalfe & Monaghan, 2001; Monaghan, 2008).

Importantly, the underlying mechanisms responsible for the phenotypic differences measured between urban and rural populations remain poorly understood. For instance, in Sprau et al. (2017), body mass variation in great tit nestlings were not explained by any of the environmental axes investigated in the study (i.e. ambient temperature, humidity, light and noise—Sprau et al., 2017). Such finding may imply either the involvement of factors different to those tested, or the combination of more environmental axes referable to site‐specific attributes (Sprau et al., 2017). Overall, despite growing interest in quantifying the effect of urbanization on offspring phenotypes, the extent to which distinct landscape elements belonging to the urban matrix affect avian growth and survival remains poorly studied. Specifically, previous studies investigated avian developmental rates and nestling differences in body mass in the context of dichotomous urban‐rural contrasts (Biard et al., 2017; Liker et al., 2008; Seress et al., 2018). Hence, such studies lacked high‐resolution insights on the association between specific attributes belonging to urban landscapes and nestling phenotypic divergences (but see de Satgé et al., 2019). A shift in focus from urban–rural contrasts to easily quantifiable and comparable urbanization measures is thus sorely needed. Consequently, the importance of using multiple spatial scales and continuous variables (rather than categorical) to describe the urban–rural gradient and assess the effects of urbanization on avian fitness has only recently been highlighted (Brans et al., 2017; Moll et al., 2019; de Satgé et al., 2019; Szulkin et al., 2020).

There is considerable evidence for nestling body mass prior to fledging to be positively correlated with post fledging survival (Nur, 1984; Tinbergen & Boerlijst, 1990). As mass is a polygenic trait of high heritability (Merilä & Sheldon, 2001), a quantification of selection patterns on that trait may be of particular relevance in human‐modified landscapes to understand the emergence of possible distinct urban ecotypes. At present, we are only aware of three studies that investigated the direction and strength of urban‐driven selection in birds (Caizergues et al., 2018; Rodewald, Shustack, & Jones, 2011; Senar, Conroy, Quesada, & Mateos‐Gonzalez, 2014): Rodewald et al. (2011) measured plumage colour in northern cardinals (*Cardinalis cardinalis*) and highlighted relaxed sexual selection on urban male colouration relative to their rural counterparts; this was mainly due to a disassociation between brightness of male plumage and urbanized landscape attributes (Rodewald et al., 2011). Senar et al. (2014) also inferred the relationship between a sexual trait—the size of the black tie in male great tits—and survival, and reported increased survival for great tits with large black ties breeding in forests (e.g. directional positive selection), and decreased survival of great tits with large black ties breeding in urban areas (directional negative selection). More recently, Caizergues et al. (2018) found that great tit males breeding in the forest had a higher reproductive success when they were leaner: interestingly, this association was not confirmed in their urban conspecifics. The authors suggested that negative selection on adult body mass might be explained by an association between parental effort (later translated into a weight loss during the feeding activity) and the number of fledglings active in the brood (Caizergues et al., 2018). Overall, this finding suggests that morphological differences between urban and rural birds may not be the result of an adaptive response driven by a recent divergent selection (Caizergues et al., 2018). The paucity of these results highlights the fact that our understanding of the strength and directionality of selection driven by urbanization is still scarce, particularly in terms of selection on juvenile traits. Therefore, more studies relating phenotypes to fitness are required to better understand patterns of natural selection across the urban matrix.

Here, data on nestling body mass and survival in two passerine species (great tits *Parus major* and blue tits *Cyanistes caeruleus*) were collected across three years and in eight study sites located within and outside the city of Warsaw, Poland. We quantified the percentage of Impervious Surface Area (ISA) around each nestbox, thus offering an easily quantifiable proxy of urbanization. ISA also covaries with a large array of urban‐driven axes (i.e. positively with temperature ‐ the urban heat island effect—Diamond and Martin, (2020), sound and light pollution, and negatively with tree cover and distance to roads—Szulkin et al., (2020)). Moreover, characterizing urbanization at the nestbox level with high‐resolution ISA data derived from remote sensors allows for straightforward comparisons of the magnitude of ISA‐driven biological effect sizes between studies. The following research questions were addressed:

1. Does urbanization affect nestling growth and body mass measured at regular age intervals?
2. Does urbanization affect nestling survival at different stages of development?
3. Is the covariation between nestling body mass and survival dependant on the level of urbanization?

We predict a pervasive, negative effect of ISA on mass at the peak of nestling food requirements (*c*. 10 days after hatching in tits). Indeed, as ISA levels rises, the proportion of green areas decrease, leading to a reduction of caterpillars available in the environment. We further test the hypothesis that increasing ISA will impact survival, as nestling body mass is a predictor of fledging success. Finally, we also quantify selection for body mass shortly after hatching and test whether the resulting effect size is larger in a high ISA environment.

## 2 MATERIALS AND METHODS

### 2.1 Study sites

**Figure 1:**
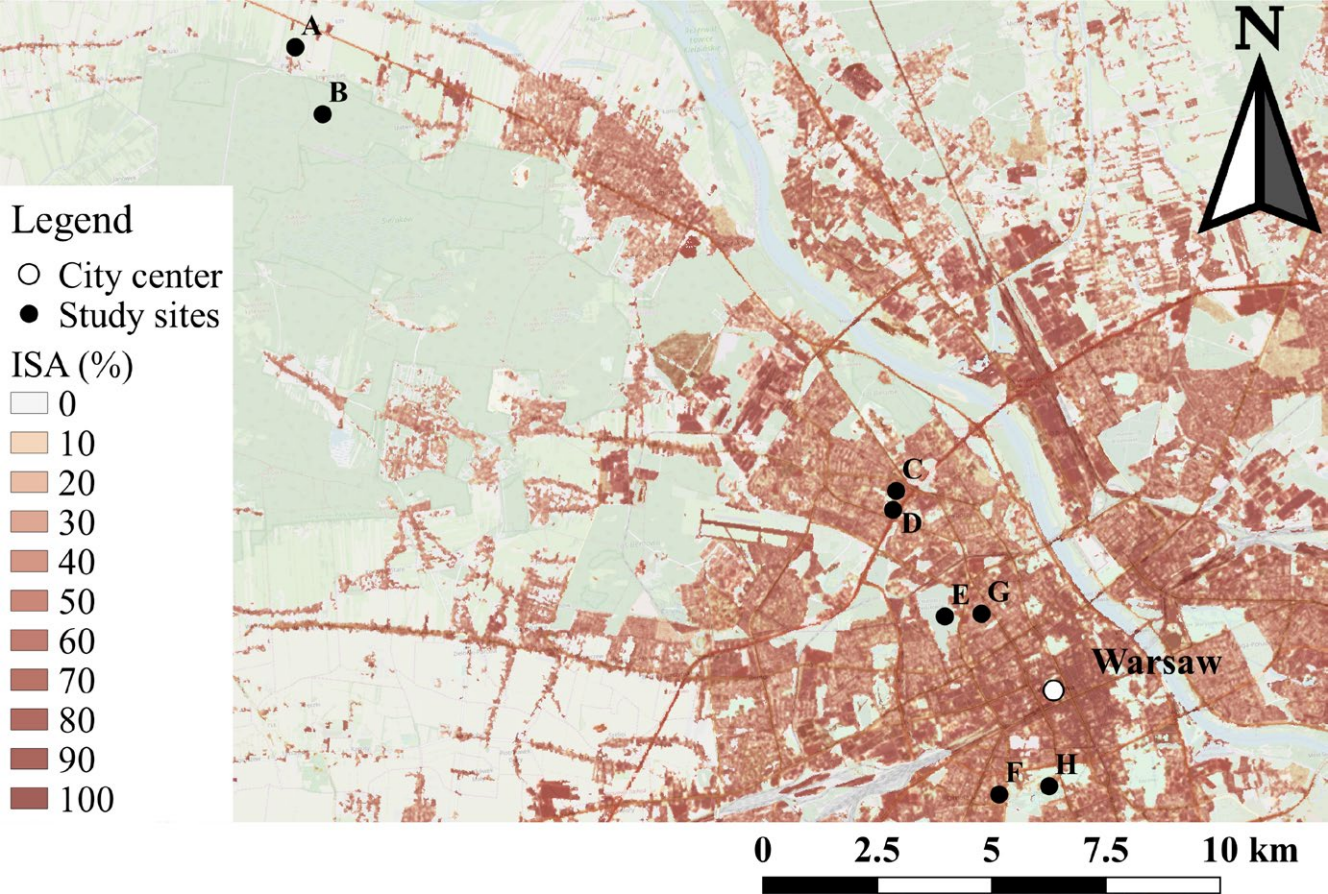
Study sites in a gradient of urbanization in the capital city of Warsaw, Poland. These include: a suburban village (a), a natural forest (b), two residential areas (c and g), two urban woodlands (d and e), an office area (f) and an urban park (h). The white dot indicates Warsaw city centre (Palace of Culture and Science). The layer visualized on the map is the original raster file used for the analyses described in section 2.2

Data were collected for three field seasons between 2016 and 2018 in a gradient of urbanization in the city of Warsaw, Poland. Five hundred Schwegler woodcrete nestboxes (type 1b with 32 mm entrance hole, suitable for great tits and blue tits) were erected in a 50 meters (m)  grid in eight contrasted study sites representative of the urban mosaic: six sites were located within the city borders while two were exurban sites (Figure 1). The total number of nestboxes within each area varied from 21 to 110. While the monitoring of three sites (B, E and H) had started in 2016, these and all other sites were monitored in 2017 and 2018. The location of all study sites, ordered based on their decreasing distance to the city centre, is presented in Figure 1 and described as follows (see also Corsini, Dubiec, Marrot, and Szulkin (2017), Corsini, Marrot, and Szulkin (2019) and Figure S1 for further details):

- Suburban village (47 nestboxes—2017, 2018). Palmiry village (20°46'48.9748" E ‐ 52°22'11.3382" N) extends for *c*. 95 hectares (ha) and is located *c*. 21 km northwest of Warsaw city borders, in proximity to Kampinos National Park. The site is characterized by residential homes with large gardens.
- Natural forest (110 nestboxes—2016, 2017, 2018). Kampinos National Park (20°47'14.3867" E ‐ 52°21'22.5409" N) is a large mixed‐coniferous forest located *c*. 20 km northwest from Warsaw city borders. It covers a surface of *c*. 38.500 ha, 15% of which is now under strict protection. The forest is characterized by a dominance of pine trees (*Pinus sp.,* 1753), followed by oaks (*Quercus sp*.).
- Residential area II (52 nestboxes—2017, 2018). Osiedle Olszyna neighbourhood (20°57'39.37097" E ‐ 52°16'23.71883" N) covers *c*. 19 ha. It is characterized by an alternation of green spaces, recreational facilities and residential buildings (blocks of flats), and is adjacent to Urban Woodland II (Las Olszyna).
- Urban woodland II (21 nestboxes—2017, 2018). Las Olszyna (20°57'33.93652" E ‐ 52°16'10.55093" N) is an urban green space composed of a deciduous, wet alder forest and an adjacent playground. The forest covers 3.4 ha and is mainly composed of common alders (*Alnus glutinosa*), birches (*Betula sp*.) and oaks (*Quercus sp*.).
- Urban woodland I (91 nestboxes—2016, 2017, 2018). The Jewish cemetery (20°58'23.44285" E ‐ 52°14'52.45584" N) became a dense forest in the post‐war period. It extends for 33 ha and is mainly characterized by a naturally regenerating habitat. With its particular landscape of moss‐covered tombstones and dense tree cover, this wild urban forest is composed of an alternation of native and exotic tree species, which mainly includes Norway maples (*Acer platanoides*), oaks (*Quercus sp*.), birches (*Betula pendula*), and elms (*Ulmus sp*.). In contrast to all the other study sites, opening hours regulate visitor access to the site.
- Office area (28 nestboxes—2017, 2018). The Warsaw University Science Campus (20°59'8.85224" E ‐ 52°12'43.77676" N) extends for *c*. 9 ha. It is situated in one of the central districts of Warsaw. The presence of offices, university buildings, canteens and dormitories provide a wide range of facilities for students.
- Residential area I (46 nestboxes—2017, 2018). The Muranów neighbourhood (20°59'5.74332" E ‐ 52°14'52.17925" N) covers *c*. 36 ha: as in residential area II, it is a typical housing estate composed of an alternation of blocks of flats and green spaces.
- Urban park (105 nestboxes—2016, 2017, 2018). Pole Mokotowskie (21°0'6.98321" E ‐ 52°12'46.66874" N) is an urban green area located in proximity to Warsaw city centre. Its alternation of flowerbeds, grass and trees covers a surface of 65 ha, offering a centrally‐located recreational site for city dwellers.

### 2.2 Quantifying urbanization

Urbanization was quantified in a 100 m radius around each nestbox in the study. This corresponds to literature‐based estimates of parental foraging while feeding nestlings, assessed in blue tits to average 53.2 m (±22.9 *SD*) in food poor (but natural) environments (Tremblay, Thomas, Blondel, Perret, & Lambrechts, 2004). In such food poor environments, birds were also reported to fly beyond 50 m from the nest in *c*. one‐third of all foraging trips (Tremblay et al., 2004). An estimation of urbanization in a 100 m radius around each nestbox thus corresponds to a conservative estimate of the range of food foraging distance covered by parents of offspring developing in the nest. Within this radius, we estimated the proportion of Impervious Surface Area (ISA) in QGIS following Szulkin et al. (2020). Specifically, a 20‐m‐pixel resolution of ISA extrapolated via satellite imagery from 2015 (Copernicus Land Monitoring Services, https://land.copernicus.eu/sitemap; see also Figure 1) was used to define ISA around each nestbox. Such index, expressed as a percentage, included all built‐up areas that replaced original natural cover or water surfaces with an artificial and usually impervious surface. These artificial surfaces include built‐up areas (such as infrastructural networks and buildings) and other elements characterized by a long cover duration. For further details on the imperviousness index description, see https://land.copernicus.eu/sitemap.

### 2.3 Life‐history data collection and nestling measurements

Starting from mid‐March, nestboxes were inspected weekly to record the date of the first egg laid and clutch size. Hatching date was determined by visiting the nest one day before the expected hatching (12 days after the last egg of the clutch was laid) and around hatching date. Both laying and hatching dates were coded by setting the 1st of April as day 1. Only first broods, defined as broods that started no later than 30 days after the very first brood in a given year and site (Van Balen, 1973), were included in the analyses. Nestlings were uniquely marked by toenails clipping or by using waterproof markers on their first measurement day. In 2016, nestlings were individually weighed every 2 or 3 days from hatching (day 1) until ringing (day 15 or exceptionally, day 14 or 16, when the brood could not be accessed on day 15). In 2017 and 2018, nestlings were weighed specifically on days 2, 5, 10 and 15. Mass was recorded to the nearest 0.1 g using digital scales (KERN pocket balance CM 150‐1N). At each nestbox visit, individual survival status (survived = 1, dead = 0) and brood size (number of chicks alive in the nest) were recorded.

In 2016, up to 50 µl blood samples were collected by puncturing the brachial vein of each 15 days old nestling. Blood samples were subsequently stored in 99.0% ethanol at + 4°C until molecular sexing analysis. Finally, nestboxes were checked *c*. 25 days after hatching to determine fledging success for each individual nestling: individuals found dead in the nest and individuals that were not present in the nestbox had a fledging success of 0 and 1, respectively.

### 2.4 Molecular sexing

Approximately 20 μl of blood was transferred to a sterile tube and centrifuged; the supernatant was removed, and the sample was dried in order to remove the ethanol. Genomic DNA from 138 blood samples was extracted with the Blood Mini kit (A&A Biotechnology) following the manufacturer's protocol, while DNA from 152 samples was extracted using the DNeasy 96 Blood & Tissue kit (Qiagen). The sex of nestlings was identified molecularly based on size differences in CHD‐linked (chromodomain helicase DNA binding protein gene) sequences from W and Z chromosomes. These sequences were amplified using primers P2 and P8 (Griffiths, Double, Orr, & Dawson, 1998) and the following PCR conditions: initial denaturation 94°C/2 min, 30 cycles, denaturation 94°C/30 s, annealing 48°C/30 s, template extension 72°C/1 min, and a final extension 72°C/5 min. Each 10 μl PCR reaction mixture contained 2 μl DNA, 1 × buffer, 1.5 mM MgCl_2_, 0.2 mM dNTP, 0.5 μM of each primer and 0.5 units of *Taq* polymerase (GoTaq G2 Hot Start Polymerase, Promega). Each plate contained a negative control (with ddH_2_O instead of DNA) to test for possible contamination. PCR products were separated by electrophoresis at 5V/cm for 40 min on a 3% agarose gel stained with Midori Green DNA Stain (Nippon) and visualized under ultraviolet light. Individuals with two bands were scored as females and with one band as males.

### 2.5 Extrapolating data on temperature at the nestbox level

Weather data over the three‐year period were provided by the Polish Institute of Meteorology and Water Management (IMGW‐PIB). Average daily temperature data were computed from Warsaw Okecie and Legionowo weather stations; the nearest sampling points for the study locations situated within and outside the city borders, respectively.

To estimate temperature experienced by growing nestlings at the nestbox level, average temperature was calculated at each nestbox across specific time periods of interest: for growth curve analyses, we used nestbox‐specific averaged temperature from day 1 to day 15 of nestling growth. For body mass and survival analyses, we used averaged temperature from day 1 to day 2, from day 2 to day 5, from day 5 to day 10 and from day 10 to day 15, which correspond to the intervals between mass measurements. Finally, for fledging success, we used averaged temperature from day 15 to day 25.

### 2.6 Statistical analyses

All analyses were conducted within the R computing environment version 3.6.2 (R Development Core Team, 2011). Functions and specific packages are further detailed below. All plots were built using the R package *ggplot2* (v. 3.1.0) (Wickham, 2011). Post‐hatching nestling growth, body mass and survival were investigated for great tits and blue tits separately, as these two passerine species differ in terms of body mass and development. Importantly, individual responses to urbanization may also vary between species.

Research questions were addressed in a three‐step process:

1. To test for an association between nestling development and a continuous change in urbanization, growth parameters were extracted from a high‐resolution temporal sampling of mass performed every 2–3 days across three study sites recorded in 2016 and compared against nestbox‐specific variation in ISA (section 2.6.1); this data set only included nestlings that survived until day 15 to generate full growth curves for these individuals. Additionally, mass measurements in 2017 and 2018 were recorded at larger time intervals (days 2, 5, 10 and 15) and were collected in eight heterogeneous sites contributing to the urban mosaic. The full data set of nestling body mass collected from 2016 to 2018 was thus used to investigate the effects of urbanization at distinct points in nestling developmental time on days 2, 5, 10 and 15, and also included nestlings that did not survive until day 15 after hatching (section 2.6.2).
2. The covariation between fitness (i.e. nestling survival) and urbanization level (i.e. ISA) was tested at consecutive developmental stages until fledging across three years (section 2.6.3).
3. Standardized selection differentials (s) on mass shortly after hatching (day 2) were estimated in low‐ and high ISA environments (section 2.6.4).

#### 2.6.1 Growth curve parameters: urbanization‐driven variation in nestling growth trajectories

High temporal resolution data of nestling body mass were collected in 2016 across three study sites (sites B (natural forest), E (urban woodland) and H (urban park) in Figure S1). To visualize ISA‐driven differences in great tit and blue tit mass gain, body mass measurements were averaged by brood and growth curves were plotted for two ISA categories: low ISA and high ISA. As great tits and blue tits may occupy nestboxes characterized by different levels of ISA in their vicinity, a median value of ISA for nestboxes corresponding to all breeding events included in the growth curve data set was derived for each species separately. Consequently, brood‐level growth curves were attributed to the low or high ISA category when below or above the median value of ISA for each species. The average ISA value for nestboxes located in high ISA corresponded to 13.3% and 16.8% in great tits and blue tits, respectively.

To assess an association between ISA and growth parameters, high temporal resolution data collected in 2016 were used to extract growth curve parameters from nestlings that (a) survived until ringing day (14, 15 or 16 days after hatching) and (b) were measured at least six times across that time frame (with the last measurement collected between day 14 and 16 after hatching). Individuals that died or disappeared before day 14 were discarded because their growth dynamics may not reflect those of nestlings that survived up to day 15 or 16. The R package *FlexParamCurve* was used to infer the best fitting growth curves and extract their parameters (updated version 1.5–5, Oswald, Nisbet, Chiaradia, & Arnold, 2012). *FlexParamCurve* uses curves of the Double Richard family, which are parametric non‐linear functions generated by the combination of two *S*‐shaped generalized logistic curves. Such function structure demonstrated to be most appropriate when describing avian growth trajectories (Arnold, Nisbet, & Oswald, 2016; Oswald et al., 2012). While the first logistic curve characterizes the monotonic (thus, unidirectional and increasing) growth stage from hatching to the moment when nestlings reach their maximum size, the second logistic curve can detect non‐monotonic mass changes, such as the period of mass recession (*i.e*. nestlings losing weight before fledging, which rarely occurs in passerines; (Arnold et al., 2016)).

Double Richard curves are defined by the equation below:

(1) $$y=\frac{\mathrm{Asym}}{{\left[1+me^{‐k(x‐Infl)}\right]}^{1/m}}+\frac{Asym^{\prime }}{{\left[1+me^{‐k^{\prime }\left(x‐Infl^{\prime }\right)}\right]}^{1/m^{\prime }}}$$

where y indicates the estimated mass at day ***x***. The remaining growth curve parameters are detailed below:

- Asymptotic mass *Asym* (projected weight at age 15–16 days).
- Inflection point *Infl* (nestling age (in days) when fastest growth occurs).
- Growth rate parameter *k* (rate at which the slope of the curve changes with age).
- Shape parameter *m* (shape of the generalized logistic curves) in the increasing curve.

while ***Asym***′, ***Infl***′, ***k***′ and ***m***′ describe the above‐mentioned parameters for the decreasing curve (Oswald et al., 2012).

The *pn.mod.compare* function in the *FlexParamCurve* package was used to automatically select the best—fitting curve (Oswald et al., 2012). The function produced a final equation that maximizes the number of individuals successfully fitted and minimizes the residuals of each fit (Oswald et al., 2012). In order to infer the best curve equation for great tits and blue tits, we used a population‐level growth selection for each species separately: the functions *pn.mod.compare* and *pn.modselect.step* in *Flexparamcurve* selected a monotonic‐standard *Richard curve* (also identified as *generalized logistic curve—*Oswald et al., 2012) as best fitting model for our target species (Table S1). Specifically, the *standard Richard curve* best described the monotonic growth of 86.4% great tit and 91.5% blue tit nestlings of our populations, and further analyses of growth curve parameter were performed only on individuals fitting this growth curve model (Table S1). Three great tit nestlings (1.2% of all tits inspected for growth curve tests) were characterized by a negative inflection point and were consequently excluded from subsequent analyses. Ultimately, only growth curve parameters extrapolated from the top ranked model were used to test whether nestling development correlates with the urbanization level.

The association between nestling growth curve parameters (*Asym, Infl* and *k*) and ISA around each nestbox, was tested using Linear Mixed Effects Models (*lmer function*, R package "*lme4*" v. 1.1–21) in a model selection framework (Barton, 2018). Each growth curve parameter was fitted as response variable and all models included the following variables as fixed effects: ISA, laying date and number of nestlings hatched in the nest (nestling development is known to be affected by brood size (Sanz & Tinbergen, 1999), sex and average temperature. Sex was included in all growth curve models to control for sex‐driven differences in nestling development often observed in tits (Morganti, Rubolini, Caprioli, Saino, & Ambrosini, 2017). Brood ID was fitted as random effect to control for the non‐independence of nestlings within the same nest. Because of collinearity between site‐level ISA and nestbox‐specific ISA, urbanization was here specifically quantified at high resolution by focusing on ISA at the nestbox level. Consequently, brood ID was fitted as random effect in all global models, but the variable “site” was not included in the models. When testing for multicollinearity, laying date revealed to be highly correlated with average temperature (see Pearson's correlation tests shown in Figure S2); thus, temperature was dropped from all global models (including the subsequent analyses performed on body mass, survival and selection differentials). As variance inflation factors (VIF) for all other explanatory variables were below 2, no issues due to multicollinearity were otherwise detected (Zuur, Ieno, Walker, Saveliev, & Smith, 2009). The global models were subsequently used to generate a set of models with all possible combinations of fixed effects (R package *MuMIn* v. 1.43.15, see Barton, 2018). Models were graded according to Akaike's information criterion (AIC_c_) to determine those with the best fit (Burnham & Anderson, 2004). Model‐averaged coefficients for a subset of models (ΔAIC_c_ < 2) were extracted. Since some Akaike weights of best models were below 0.9 and therefore high model selection uncertainty existed, full‐model averaging was used (Grueber, Nakagawa, Laws, & Jamieson, 2011; Symonds & Moussalli, 2011). Upper and lower bounds of the 95% confidence intervals (CI) were calculated for each parameter.

#### 2.6.2 Association between nestling body mass and urbanization

To assess the impact of urbanization (quantified as the percentage of ISA around each nestbox) as a driver of nestling body mass variation, model fitting included three years of data and focused on individual weight measurements recorded when nestlings were 2, 5, 10 and 15 days old. For each analysis, the data set included nestlings that were alive at the corresponding stage of development. Linear mixed models (LMMs) were run with Gaussian distribution (“*lmer*” function in the R package "*lme4*"—Bates, Mächler, Bolker, & Walker, 2014) in a model selection framework as described in 2.6.1 (Barton, 2018). The following explanatory variables were included in all models: ISA, laying date, number of nestlings (fitted as the number of nestlings alive in the nest at each respective weighing day) and year (as categorical variable, to quantify possible year‐driven differences (Bolker et al., 2009)). Brood ID was fitted as random factor.

#### 2.6.3 Association between nestling survival and urbanization

To test whether survival (0/1) of great tit and blue tit nestlings is dependent on ISA, generalized linear mixed models (GLMMs) were run with binomial distribution and *logit‐link function* (“*glmer*” function in the *R package "lme4"*—Bates et al., 2014) in a model selection framework as described in 2.6.1. To avoid problems with model convergence, continuous predictor variables were standardized using z‐score standardization before model running to achieve a mean of zero and a standard deviation of 1. The effect of ISA on survival was quantified across independent age categories: survival was thus defined as early‐stage survival (e.g. whether a nestling survived between day 2 and day 5 after hatching), medium‐stage survival (between day 5 and day 10 after hatching), late‐stage survival (between day 10 and day 15 after hatching) and fledging survival (between day 15 and day 25 after hatching). Within each model, the following explanatory variables were included: ISA, laying date, number of nestlings (as found in the nest at the start of each respective age category) and year. Nestling non‐independence was controlled by including brood ID as random factor.

#### 2.6.4 Selection differentials in contrasted urban environments

Similarly to earlier studies comparing selection differentials in contrasted habitats (i.e. urban versus rural environment, see Caizergues et al., 2018), we inferred the intensity of selection on body mass in contrasted ISA environments. Specifically, standardized univariate selection differentials *s*, expressed as the covariance between fitness *w* and trait *z* (Lande & Arnold, 1983): s = Cov[w,z], were calculated in low and high ISA environments separately. Focus was given to mass on day 2 as it is a polygenic trait likely to carry a considerable genetic component and is also the earliest available phenotypic measurement before mortality removes parts of variation in that trait. Indeed, all later life‐history stages are marked by selective mortality, which likely removes individuals of lowest body mass from the dataset as chicks often die from starvation. Consequently, records of mass are increasingly likely to be impacted by selective mortality over time. Breeding events from the full dataset (2016–2018, *n* = 328) were assigned to low or high ISA environments if any given nestbox ISA value was below or above the median ISA value of all breeding events, calculated for each species separately.

Estimates of standardized selection differentials were obtained from GLMMs as detailed in section 2.6.3. Fitness *w* was computed as individual (chick‐level) survival at fledging (a binomial trait), standardized by yearly average survival for the population. All response variables were z‐standardized at an annual level, and included: trait z, corresponding to mass at day 2, laying date and number of nestlings recorded at day 2. Brood ID was fitted as random effect in the models.

To test for a difference in selection operating in high and low ISA environments, low and high ISA datasets were combined and the interaction term ISA environment (low or high) * trait (mass day 2) was added as fixed effect, while all other fixed and random effects were included as above.

## 3 RESULTS

A total of 2,700 chicks from 328 broods were followed across three years, composed of 143 great tit broods (1,118 chicks) and 185 blue tit broods (1,582 chicks).

### 3.1 Growth curve parameters: urbanization‐driven variation in nestling growth trajectories

**Figure 2:**
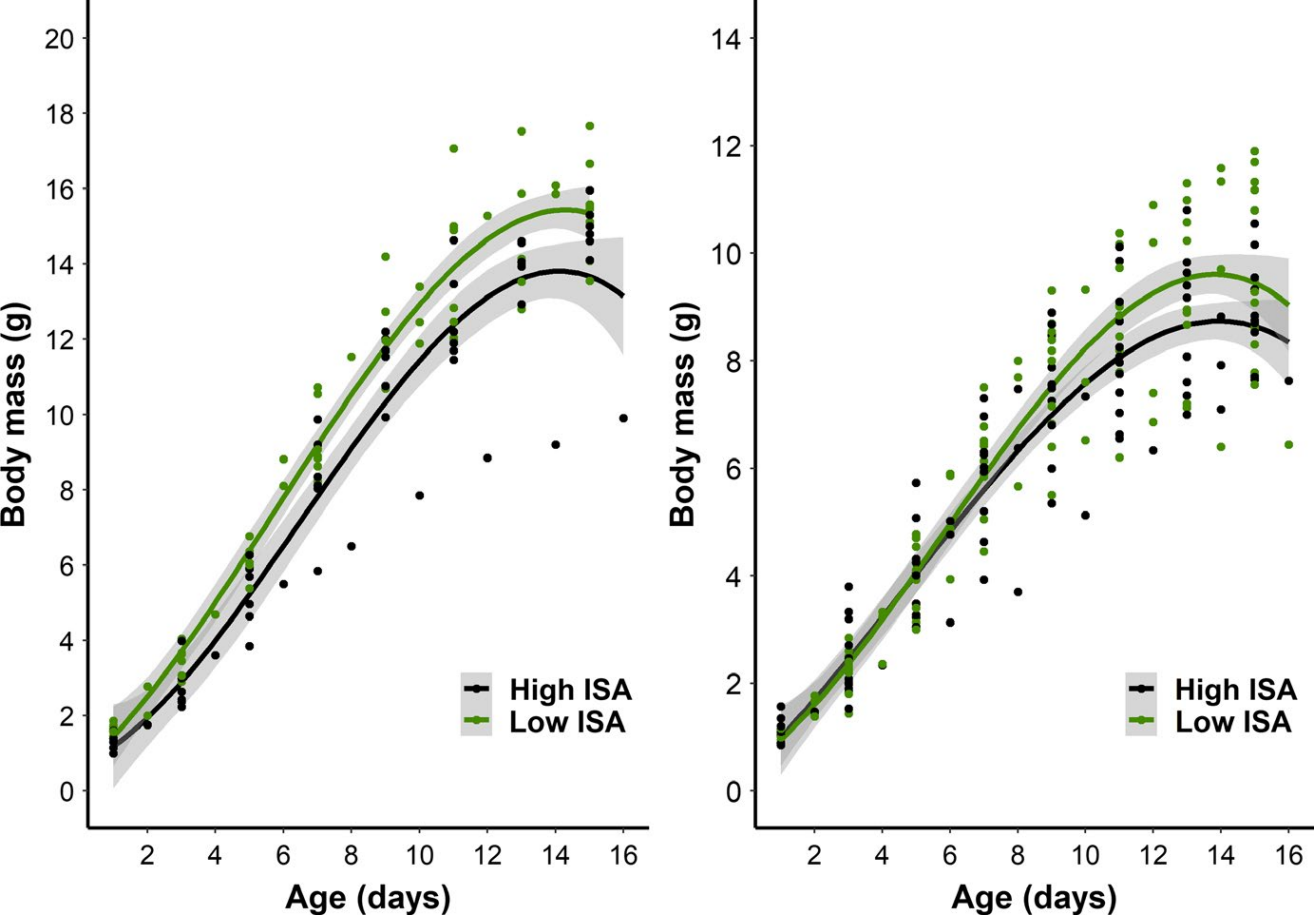
Nestling growth curves in high (black) and low (green) ISA environments for great tits (left panel) and in blue tits (right panel). Only nestlings that survived until day 15 (or day 14 or 16 in the case of a few broods, which could not be accessed on day 15) were included in this visualization. Dots refer to original mass measurements averaged by brood to control for non‐independence. Curves were drawn using "*method = 'gam'*, *formula = y ~ poly (x, 3)*" in *ggplot2*. For visual clarity, ISA was categorized as low and high, specifically: low ISA—mean of 0.64% ISA for great tits and 1.71% ISA for blue tits; high ISA—mean of 13.3% ISA for great tits and 16.83% ISA for blue tits; (*n* = 15 great tit broods and *n* = 27 blue tit broods)

Growth curves of nestlings that survived until 15 days of age in low and high ISA environments are visually distinct (Figure 2). The age of fastest growth, characterized by the inflection point, increased with increasing ISA around each nestbox in blue tits: thus, growth slowed down later in areas with a greater proportion of impervious surface (Table 1). However, ISA was not kept in the averaged models for other growth curve parameters (Table 1, Table S2). Thus, ISA did not play a major role on asymptotic mass or growth rate in the dataset of birds that survived until day 15. Although the variables “sex” and “number of nestlings” were both retained in the average models (Table 1), significant differences were found only for nestling sex, confirming that male nestlings were heavier in terms of asymptotic mass relative to females in both species (Table 1).

**Table 1:** Model‐averaged summary statistics of best fitting LMMs testing the effect of ISA on great tit and blue tit growth curve parameters

Response	Species	Variable	Estimate	*SE*	CI 95%	Relative importance
Great tit	**Asymptotic mass (g)**	(Intercept)	14.268	2.031	10.286; 18.249	
(*n* nestlings = 67)		**Sex (male)**	**2.027**	**0.590**	**0.869; 3.184**	**1.00**
(*n* broods = 15)		*N* nestlings	0.380	0.303	−0.215; 0.974	0.34
	**Inflection point (days)**	(Intercept)	5.520	0.383	4.743; 6.291	
	**Growth rate (k)**	(Intercept)	0.389	0.065	0.257; 0.520	
Blue tit	**Asymptotic mass (g)**	(Intercept)	10.404	1.540	7.386; 13.423	
(*n* nestlings = 144)		**Sex (male)**	**1.001**	**0.258**	**0.496; 1.507**	**1.00**
(*n* broods = 26)		*N* nestlings	−0.278	0.159	−0.590; 0.034	0.38
	**Inflection point (days)**	(Intercept)	5.669	0.429	4.827; 6.511	
		**ISA**	**0.071**	**0.029**	**0.014; 0.127**	**0.305**
	**Growth rate (k)**	(Intercept)	0.662	0.103	0.460; 0.863	
		Sex (male)	−0.172	0.088	−0.343; 0.001	0.34

Note

Model‐averaged summary statistics of Linear Mixed Models (LMMs) testing the effect of ISA on great tit and blue tit nestling growth. Growth curve parameters were extrapolated from high‐resolution data collected in 2016 and included Asymptotic mass, Inflection point and Growth rate. Each growth curve parameter was fitted as response variable with a Gaussian distribution. All global models included the following predictors: ISA (percentage of built‐up area measured at the nestbox level), egg laying date (1 = 1st of April), number of nestlings (“*N* Nestlings,” hatched in the nest) and sex (the effect of sex is reported for males relative to females). Brood ID was fitted as random effect. Parameters with confidence intervals not overlapping 0 are highlighted in bold.

### 3.2 ISA‐driven effects on nestling body mass

For both species, none of the originally selected variables were retained in the average model of nestling body mass 2 days after hatching (Table S3, Table 2), thus confirming that body mass shortly after hatching is not different across the entire urbanization gradient. However, the impact of ISA on body mass was consistently negative for both species later in development (Table 2): ISA was retained in the best models of nestling body mass variation 5 and 10 days after hatching for great tits and 10 and 15 days after hatching for blue tits (Table S3, Table 2). Additionally, brood size (number of nestlings) positively affected great tit nestling body mass 15 days after hatching (Table S3, Table 2). In blue tits, body mass 10 days after hatching also increased if chicks hatched later in the season (Table 2). Overall, these results confirm that in both species, while body mass does not differ at hatching, body mass differences accumulate in a gradient of ISA over time.

**Table 2:** Model‐averaged summary statistics of best fitting LMMs for great tit and blue tit nestling body mass variation at consecutive developmental stages

Species	Response	Variable	Estimate	*SE*	CI 95%	Relative importance
Great tit	**Mass day 2** (*n* = 869)	Intercept	1.981	0.037	1.909; 2.054	
	**Mass day 5** (*n* = 928)	Intercept	5.737	0.232	5.287; 6.188	
		**ISA**	**−0.022**	**0.005**	**−0.032; −0.012**	**‐**
		Year				**‐**
		Year 2017	−0.452	0.271	−0.979; 0.075	
		Year 2018	0.104	0.259	−0.398; 0.606	
	**Mass day 10** (*n* = 732)	Intercept	11.324	1.140	9.113; 13.534	
		**ISA**	**−0.062**	**0.012**	**−0.085; −0.039**	**‐**
		Year				**‐**
		Year 2017	0.831	1.184	−1.464; 3.126	
		Year 2018	1.860	1.174	−0.415; 4.135	
	**Mass day 15** (*n* = 665)	Intercept	13.001	0.564	11.897; 14.107	
		***N* nestlings**	**0.301**	**0.083**	**0.138; 0.463**	**‐**
Blue tit	**Mass day 2** (*n* = 1,144)	Intercept	1.443	0.023	1.397; 1.489	
	**Mass day 5** (*n* = 1,295)	Intercept	3.665	0.144	3.383; 3.947	
		Year				**‐**
		Year 2017	−0.289	0.165	−0.612; 0.033	
		Year 2018	0.127	0.169	−0.204; 0.457	
	**Mass day 10** (*n* = 924)	Intercept	5.771	0.738	4.343; 7.198	
		**ISA**	**−0.029**	**0.007**	**−0.042; −0.016**	**‐**
		**Laying date**	**0.077**	**0.024**	**0.030; 0.125**	**‐**
		Year				**‐**
		Year 2017	1.022	0.614	−0.166; 2.210	
		Year 2018	1.627	0.587	0.492; 2.762	
	**Mass day 15** (*n* = 839)	Intercept	9.342	0.234	8.887; 9.797	
		**ISA**	**−0.030**	**0.008**	**−0.046; −0.014**	**‐**
		Year				**‐**
		**Year 2017**	**1.002**	**0.303**	**0.413; 1.591**	
		**Year 2018**	**0.812**	**0.302**	**0.224; 1.400**	

Note

Model‐averaged summary statistics of linear mixed models (LMMs) explaining variation in nestling body mass tested at consecutive developmental stages (2, 5, 10 and 15 days after hatching). Estimates of coefficients and unconditional standard error (*SE*), lower and upper limits of the 95% confidence intervals (CI 95%) are reported. In this analysis, no model averaging was performed as ΔAIC_c_ < 2 always identified a single model. Individual body mass 2, 5, 10 and 15 days after hatching was fitted as response variable with Gaussian distribution. All global models included the following predictors: ISA (percentage of built‐up area measured at the nestbox level), egg laying date (1 = 1st of April), number of nestlings (“*N* nestlings” that were alive in the nest while recording individual body mass) and year (2016, 2017 and 2018). Brood ID was fitted as random factor in all models. Parameters with confidence not overlapping 0 are highlighted in bold.

### 3.3 ISA‐driven effects on nestling survival

A pervasive, negative association between ISA and nestling survival was detected at distinct, independent developmental stages of great tits and blue tits (Table 3).

**Table 3:** Model‐averaged summary statistics of best fitting GLMMs of great tit and blue tit nestling variation in survival at consecutive stages of development

Species	Response	Variable	Estimate	*SE*	CI 95%	Relative importance
Great tit	Early age survival	(Intercept)	9.545	1.061	7.465; 11.624	
	(*n* = 1,104)	Laying date	1.367	0.842	−0.283; 3.017	0.75
		*N* nestlings	0.439	0.591	−0.720; 1.598	0.19
		ISA	−0.087	0.588	−1.239; 1.064	0.15
	Medium age survival	(Intercept)	4.630	1.395	1.895; 7.364	
	(*n* = 988)	**ISA**	**−1.397**	**0.484**	**−2.345; −0.449**	**1.00**
		Year				0.64
		Year 2017	0.448	1.314	−2.127; 3.022	
		Year 2018	2.082	1.232	−0.333; 4.496	
		Laying date	−0.306	0.481	−1.250; 0.637	0.16
		*N* nestlings	−0.151	0.399	−0.933; 0.632	0.14
						
	Late age survival	(Intercept)	4.688	0.774	3.170; 6.206	
	( *n* = 855)	**ISA**	**−1.466**	**0.457**	**−2.362; −0.570**	**1.00**
		Laying date	0.791	0.497	−0.183; 1.765	0.65
		*N* nestlings	−0.209	0.454	−1.098; 0.681	0.19
	Fledging survival	(Intercept)	8.877	1.286	6.357; 11.397	
	(*n* = 665)	*N* nestlings	0.675	0.652	−0.603; 1.954	0.26
		Laying date	0.265	0.733	−1.171; 1.701	0.16
		ISA	0.109	0.749	−1.359; 1.577	0.16
Blue tit	Early age survival	(Intercept)	6.034	0.868	4.332; 7.735	
	(*n* = 1,568)	Year				1.00
		**Year 2017**	**−2.208**	**0.802**	**−3.779; −0.636**	
		Year 2018	0.127	0.862	−1.562; 1.817	
		***N* nestlings**	**0.525**	**0.243**	**0.048; 1.002**	**1.00**
		ISA	−0.435	0.286	−0.996; 0.127	0.54
	Medium age survival	(Intercept)	3.263	0.642	2.005; 4.520	
	(*n* = 1,456)	**ISA**	**−0.765**	**0.272**	**−1.298; −0.233**	**1.00**
		***N* nestlings**	**−0.512**	**0.247**	**−0.996; −0.029**	**1.00**
		Year				1.00
		Year 2017	−0.950	0.797	−2.512; 0.612	
		Year 2018	0.864	0.755	−0.616; 2.343	
		Laying date	−0.521	0.293	−1.094; 0.052	0.64
	Late age survival	(Intercept)	2.548	0.464	1.639; 3.457	
	(*n* = 1,195)	**ISA**	**−0.740**	**0.368**	**−1.461; −0.018**	**1.00**
		**Laying date**	**0.714**	**0.364**	**0; 1.427**	**1.00**
		*N* nestlings	−0.151	0.341	−0.819; 0.517	0.29
	Fledging survival	(Intercept)	3.470	0.809	1.885; 5.055	
	(*n* = 838)	Year				1.00
		**Year 2017**	**2.818**	**0.961**	**0.934; 4.702**	
		Year 2018	−0.105	0.829	−1.731; 1.520	
		*N* nestlings	0.447	0.322	−0.184; 1.078	0.48
		ISA	−0.276	0.338	−0.938; 0.386	0.26
		Laying date	−0.208	0.391	−0.975; 0.559	0.22

Note

Generalized linear mixed models (GLMMs, with binomial error distribution) of variation in nestling and fledging survival in great tits and blue tits: estimates of coefficients and standard errors (s.e.), lower and upper limits of the 95% confidence intervals (CI 95%) and relative importance values of model parameters are reported. Survival was fitted as binomial response (1/0) variable for each age interval. The following variables were fitted as fixed effects: ISA (percentage of built‐up area measured at the nestbox level), laying date (“Laying date,” 1 = 1st of April), number of nestlings (“*N* nestlings,” as the total number of nestlings alive in the nest at the start of each age interval), year (study years 2016, 2017, 2018). Brood ID was fitted as random effect in all models. Parameters with confidence not overlapping 0 are highlighted in bold.

*Early stage of development (*2–5* days after hatching)*—in great tits, although the averaged model included ISA, number of nestlings and laying date (Table S4), all confidence intervals overlapped zero. In blue tits, the averaged model included ISA, number of nestlings and year of study: survival until day 5 increased with increasing number of nestlings in the brood and was year‐dependent; the confidence intervals for ISA included zero (Table S4, Table 3).

*Medium (*5–10 days*after hatching) and late stages of development (*10–15* *days after hatching*)*—in both species, ISA was retained in the averaged models (Table S4) and confidence intervals confirmed a strong negative association between nestling survival and ISA at both developmental stages. Moreover, blue tit nestling survival increased with decreasing brood size (medium age survival) and with delayed laying date (late age survival, Table 3).

*Fledging stage of development (15 days until fledging)*—in both species, all explanatory variables were retained in the averaged models except for year in the case of great tits (Table S4). However, only confidence intervals of the “year” variable in blue tits did not include zero and indicated high survival in the year 2017 (Table 3).

### 3.4 ISA‐specific selection differentials

Because of species‐specific nestbox occupancy across sites, ISA distribution across low and high ISA categories varied between species. For each species, the same number of broods was assigned to either low or high ISA category. Great tits breeding in low and high ISA environments were surrounded by an average of 0.99% and 25.85% ISA, respectively. Similarly, blue tits breeding in low and high ISA environments were surrounded by an average of 2.12 and 27.22% of ISA, respectively.

**Figure 3:**
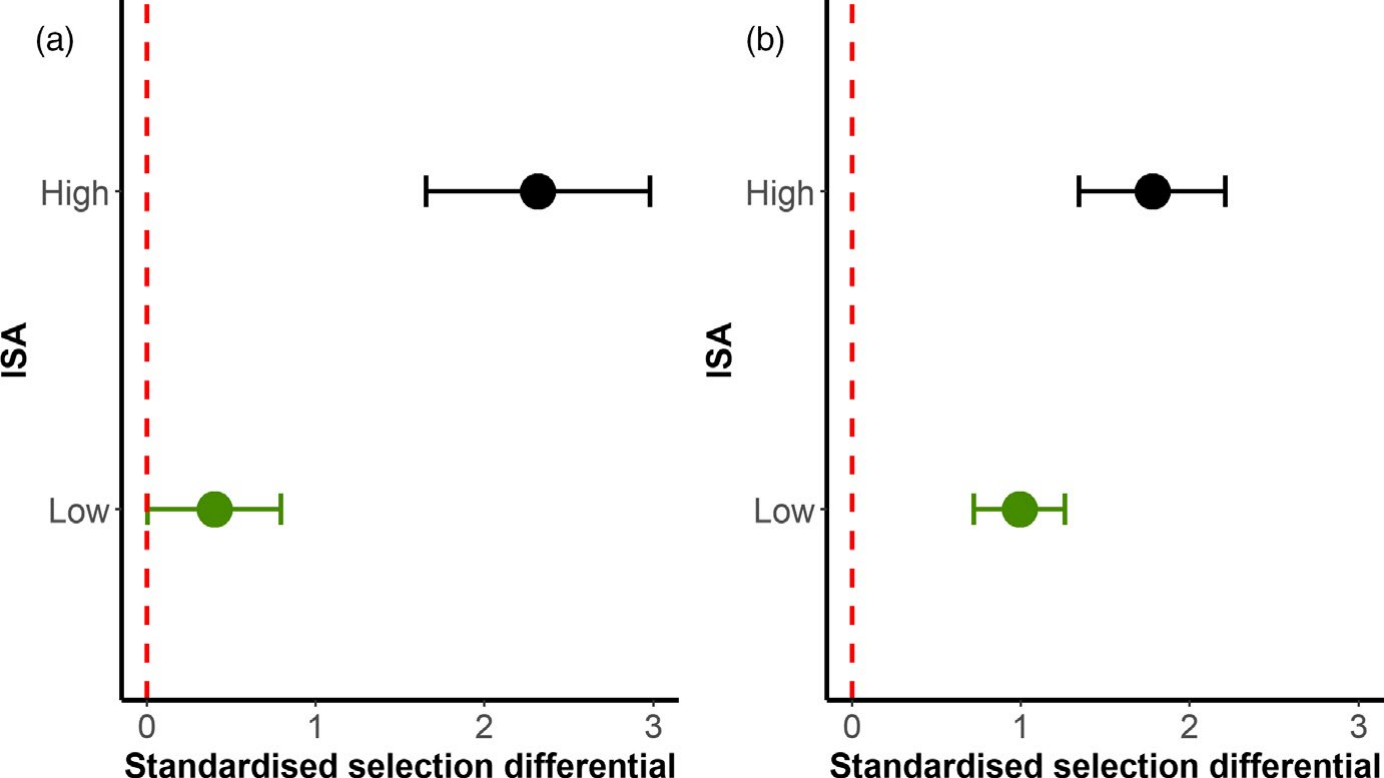
Standardized selection differentials (± standard error) on mass shortly after hatching (day 2) in low and high ISA environments for great tits (left panel) and blue tits (right panel). Fitness is computed as survival at fledging. Both the response variable (fitness) and all explanatory, continuous variables were standardized at a yearly level. ISA values were categorized as low ISA: mean of 0.99% ISA for great tits and 2.12% ISA for blue tits; high ISA: mean of 25.85% ISA for great tits and 27.22% ISA for blue tits. Selection differentials confirm positive selection on mass shortly after hatching, and the strength of selection on mass increases with increasing levels of ISA in great tits

Positive selection differentials were recorded for mass at day 2, confirming that mass shortly after hatching significantly impacts the survival of both blue tits (for high and low ISA environments) and great tits (in high ISA environment) (Figure 3; Table S5). While point estimates of selection differentials were consistently higher in a high ISA environment in both species (Figure 3), the selection differential*ISA category interaction was only significant in great tits (Table S6). These results thus confirm positive selection for heavier nestlings at birth. Importantly, the strength of selection increases in a more transformed, high ISA environment relative to a low ISA environment (Figure 3, Table S5).

## 4 DISCUSSION

This study confirms the negative and pervasive effects of ISA on nestling development, body mass and survival in two common passerine species. We also report a strong positive association between mass at day 2 (a trait expected to have a strong genetic basis (Merilä & Sheldon, 2001)) and survival at fledging, leading to consistently positive directional selection differentials on mass at day 2 in both species. Importantly, this relationship is stronger in high ISA environments (Figure 3). The study also demonstrates that the extent of Impervious Surface Area (ISA), computed around a key point of interest (such as a breeding event), is an easily computable indicator of urbanization that can be quantified at high spatial resolution. We provide a detailed discussion for each of our main findings in three distinct sections, presented below.

### 4.1 Effect of urbanization on great tit and blue tit nestling development

High temporal resolution measurements recorded in 2016 revealed that great tit and blue tit populations were mostly characterized by a monotonic growth, as commonly reported for passerine species (Arnold et al., 2016; Kunz & Ekman, 2000; Mainwaring & Hartley, 2016; Morganti et al., 2017; Remacha, Delgado, Bulaic, & Pérez‐Tris, 2016). Growth curves of birds reared in low and high ISA were markedly distinct (Figure 2). To the extent of our knowledge, this is the first study reporting that high levels of imperviousness in the environment, measured at the nestbox level, can have far‐reaching consequences on avian growth, explicitly in terms of age of fastest growth (inflection point parameter). Indeed, a 50% increase in ISA resulted in 3.6 days of delay for the time when fastest growth occurred in blue tits. The absence of a significant association between ISA and inflection point in great tits may be caused by power limitations, as there were *c*. two times fewer great tit broods than blue tit broods in that year.

Such important delay in the age of fastest growth may be related to dietary requirements: tit nestlings require a specific arthropod diet to develop uniformly during the nestling period (Naef‐Daenzer, Naef‐Daenzer, & Nager, 2000). Numerous studies carried out in natural environments emphasized the importance of food abundance on passerine nestlings growth and body mass (Perrins, 2008; Van Balen, 1973). Such findings were similarly confirmed with experimental approaches, which measured a positive association between food availability and offspring growth (Smith & Arcese, 1988). Yet, as urban growth translates into a loss of green spaces with fields and woodlands replaced by concrete and other impervious surfaces (Shaw, Chamberlain, & Evans, 2008), arthropods diversity and availability within the urban space are also compromised. Importantly, evergreen and exotic plants reduce the abundance and quality of prey items in cities (Southwood, 1961). Arthropod decline in the urban environment is also likely to be driven by environmental pollution (i.e. traffic emissions, Summers‐Smith, 2005). In addition, breeding adults need to cope with new and often challenging urban environmental conditions, which might altogether alter their foraging capacities.

### 4.2 Effect of urbanization on nestling body mass and survival at consecutive stages of development

Globally, nestling body mass recorded during three breeding seasons was strongly and negatively associated with the percentage of ISA in their immediate rearing environment. In particular, the association between nestling body mass and ISA levels was consistently negative in 5 and 10 days old great tits and in 10 and 15 days old blue tits. In parallel to this result, it was also observed that for both species, the survival of nestlings in mid and late development (between day 5 and day 10, and day 10 and day 15) was negatively associated with the extent of ISA. Interestingly, survival after 15 days of age did not depend on ISA level, suggesting that ISA‐driven selective mortality takes place at the age of maximum food demand, which in great tits and blue tits occurs around 10 days after hatching (Van Balen, 1973). While in natural environments, caterpillars are the main food source in great tit and blue tit nestlings (Perrins, 2008), their availability decreases drastically within the urban matrix (Pollock, Capilla‐Lasheras, McGill, Helm, & Dominoni, 2017). Our study shows that the more the natural stands (e.g. able to sustain caterpillar development) are replaced by high ISA in the urban space, the more the body mass of developing great tits and blue tits is reduced: thus, high levels of ISA in the nestbox vicinity increase nestling mortality. Urbanization‐driven reductions in food availability are likely to lead to higher rates of nestling mortality related to starvation: earlier studies comparing great tit and blue tit nestlings reared in private gardens with those developing in woodlands reported how the former suffered from higher mortality rates relative to the latter (Cowie & Hinsley, 1988; Lack, 1955). Moreover, the abundance, richness and size of taxa such as caterpillars, beetles, flies and spiders also tend to decrease with increasing pollution within cities (McIntyre et al., 2001; Raupp, Shrewsbury, & Herms, 2010; Shochat, Lerman, Katti, & Lewis, 2004; Zvereva & Kozlov, 2010). Food shortage can also result from the patchy structure of the urban habitat. Indeed, in cities, trees and shrubs are usually distributed in small and distant patches that are not suitable for efficient foraging for birds (Mackenzie, Hinsley, & Harrison, 2014). Other reasons for lower body mass and higher mortality in high ISA environments can include, as mentioned in *Section 4.1*, the presence of exotic tree species (normally implanted in private gardens and urban parks): indeed, in contrast to common phenological events in native plants, these may leaf and flower at different time intervals during the year, thus reducing the food base required by developing passerine nestlings. As herbivorous insects synchronize their reproduction with bud burst (Buse & Good, 1996; zowski, Szulkin, & Sheldon, 2015), there is a higher chance that a mismatch occurs between the nestling period and the peak abundance of the most important prey items in cities (Mackenzie et al., 2014).

**Figure 4:**
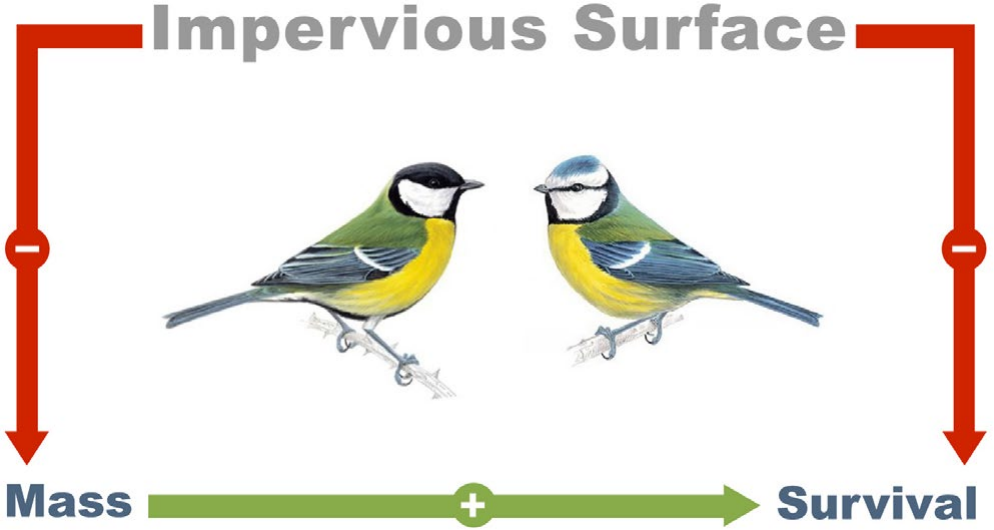
Urbanization, measured as the proportion of Impervious Surface Area around a point of interest, negatively impacts body mass and survival in 2 developing passerine birds. At the same time, body mass shortly after hatching positively increases survival till fledging, particularly in high ISA environments

As model selection identified a clear‐cut negative effect of ISA on mass variation in great tits and blue tits during the nestling period (most intensely at the peak of food demand), the same statistical framework revealed important negative effects of ISA on survival. Given the strong negative relationship between ISA and survival, and between ISA and mass (Figure 4, Table S7), the observed pattern of decreased survival in high ISA environment is likely to be mediated by food restriction at the nestling development stage along with a reduction in terms of nestling mass in high ISA environments.

### 4.3 ISA enhances selection on body mass shortly after hatching

This study is one of the very few readily reporting the strength and directionality of selection by inferring the covariation of fitness (individual fledging success) with a phenotypic trait (body mass on day 2 after hatching). Overall, the analysis of selection differentials acting on nestlings reared in nestboxes from low ISA and high ISA environments revealed a positive association between mass measured at day 2 and avian fitness in all ISA categories (Figures 3 and 4). Importantly, positive selection on mass was considerably stronger in great tits and blue tits reared in high ISA environments (Figure 3), though not significantly so in blue tits. Thus, nestlings recorded as heavy shortly after hatching were considerably more likely to fledge the nest relative to lighter nestlings, and particularly so in a high ISA environment. Our findings thus complement selection analyses led by Caizergues et al. (2018), whose study did not confirm a role for reproductive selection in explaining life‐history and adult phenotypic differences between urban and rural breeding great tits. Urban long‐term monitoring is thus required to confirm, or refute response to selection over time and across an individual's life cycle (Merilä, Sheldon & Kruuk, 2001).

A study by Liker et al. (2008) reported that differences in adult body mass between urban and rural house sparrows, with the former lighter than the latter, remained significant when birds were kept in aviaries with *ad libitum* food provided. It is unclear however whether the observed smaller body size and condition of adult urban sparrows are caused by limited food resources in adults, by limited food resources in chicks that led to smaller structural size, or because of inherent differences in the genetic makeup of these urban birds. The same authors suggest that adult body mass differences between rural and urban populations may originate earlier in life, at the nestling development stage (Liker et al., 2008). In this study, we report that, for both blue tits and great tits, (a) selection for larger body mass shortly after hatching is stronger in high ISA areas (Figure 3) and (b) nestling survival between day 5 and 10 and also between day 10 and day 15 after hatching decreases with increasing ISA. Thus, as a consequence of response to selection, a phenotypic shift for heavier nestlings at birth could be expected in environments characterized by higher levels of impervious surface.

## 5 CONCLUSIONS

This study brings to light new evidence for a pervasive and negative impact of urbanization on the evolutionary ecology of passerine developmental rates and brings unique insight into the adaptive potential of urban‐dwelling organisms. Nestbox‐specific quantification of ISA allowed for the modelling of urbanization on a continuous scale and at high spatial resolution, which consequently allowed for a finer‐grained quantification of urban environments relative to dichotomous splits such as natural/ urban environments (Szulkin et al., 2020). Consequently, an ISA‐centred perspective of urbanization can generate detailed insight into the effects of cities on avian development. Importantly, this study shows compelling evidence for positive, directional selection on mass measured shortly after birth, which is further magnified in high‐ISA environments. This is at odds with frequently reported smaller phenotypic values for tarsus and mass in nestlings and adults measured in the urban space (Caizergues et al., 2018; Chamberlain et al., 2009; Sprau et al., 2017). More generally, urban long‐term data across multiple cities is required to better understand both patterns of selection and response to selection on phenotypic and life‐history traits (Merilä et al., 2001; Santangelo et al., 2020). As the quantification of genetic and environmental components of trait variation in urban organisms requires further investigation, it is also expected that quantitative genetic inference of urban organisms will grow in the near future as the number and duration of long‐term studies in the urban space will increase. Equally important, a clearer understanding of dispersal patterns from and towards urban areas is to date limited, and further work synthesizing the generality of avian urban ecotypes and their dispersal would be valuable to draw further light on urban‐driven phenotypic and genetic variation. Further work is needed to quantify selection over time and to assess the relative role of directional and stabilizing selection on mass across successive developmental stages.

## Supporting information

Supplementary Material

## Data Availability

Data for this study are available at the Dryad Digital Repository https://doi.org/10.5061/dryad.xgxd254dp.
