# Supplementary material for: Growing in the city: Urban evolutionary ecology of avian growth rates
Source: Evol Appl. 2020 Sep 17;14(1):69–84. doi: 10.1111/eva.13081 (PMC7819560; doi:10.1111/eva.13081)
Supplement: Supplementary file 1 — Supplementary Material [file EVA-14-69-s001.docx]

**Supplementary Material**

**Figure S1. Satellite imagery of each study site.**


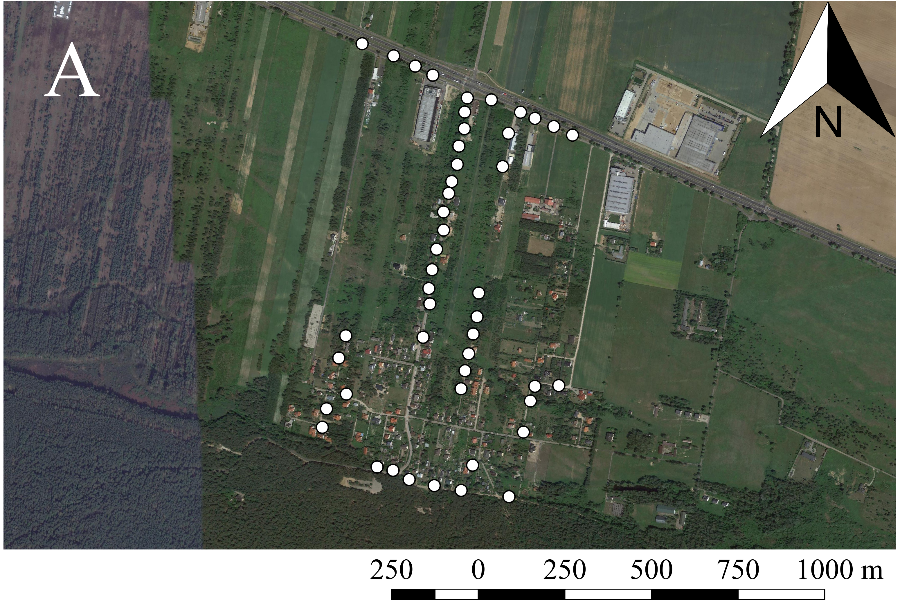

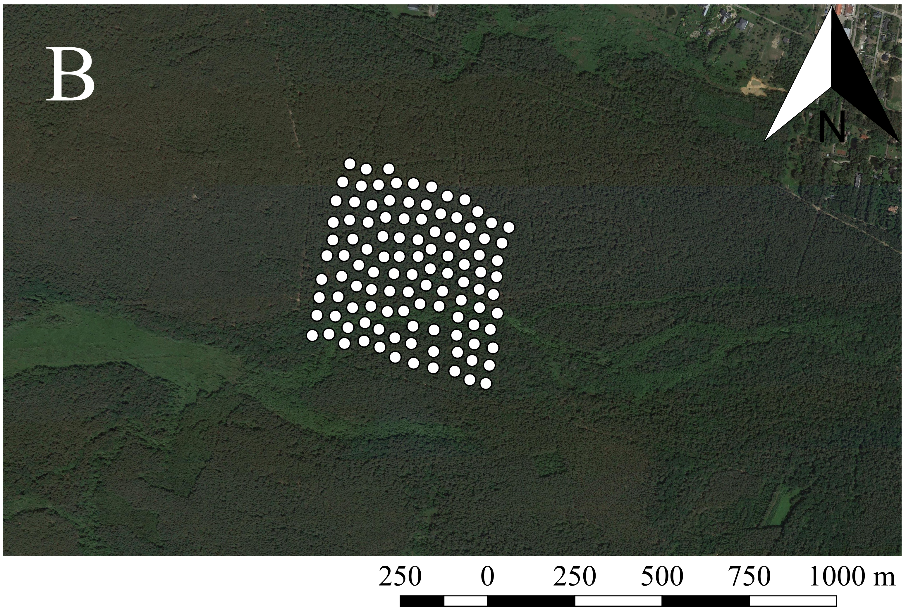

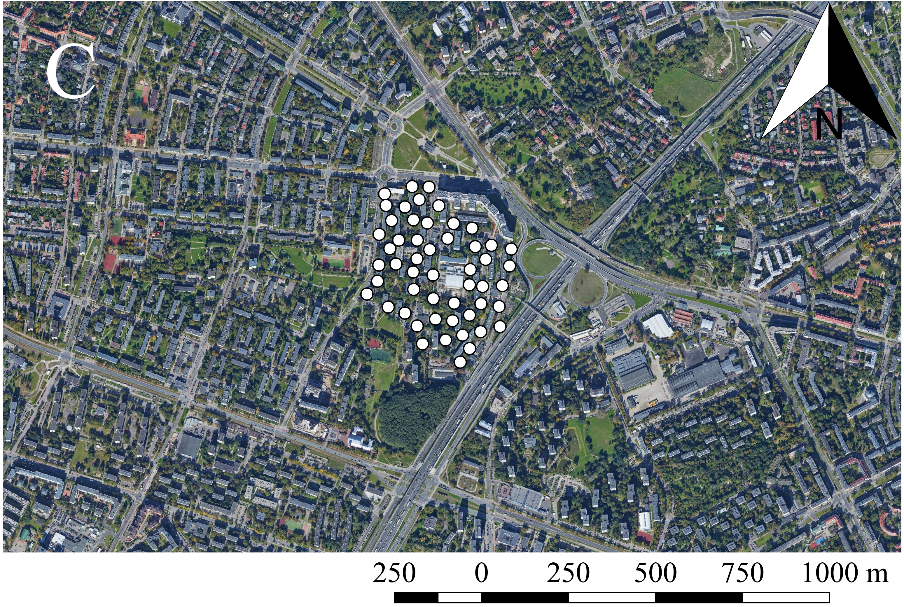

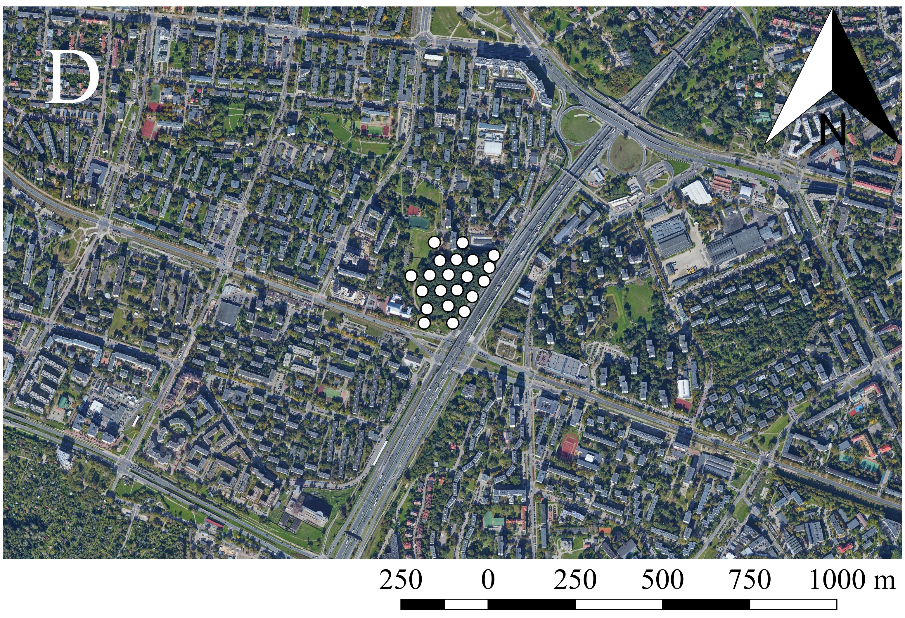

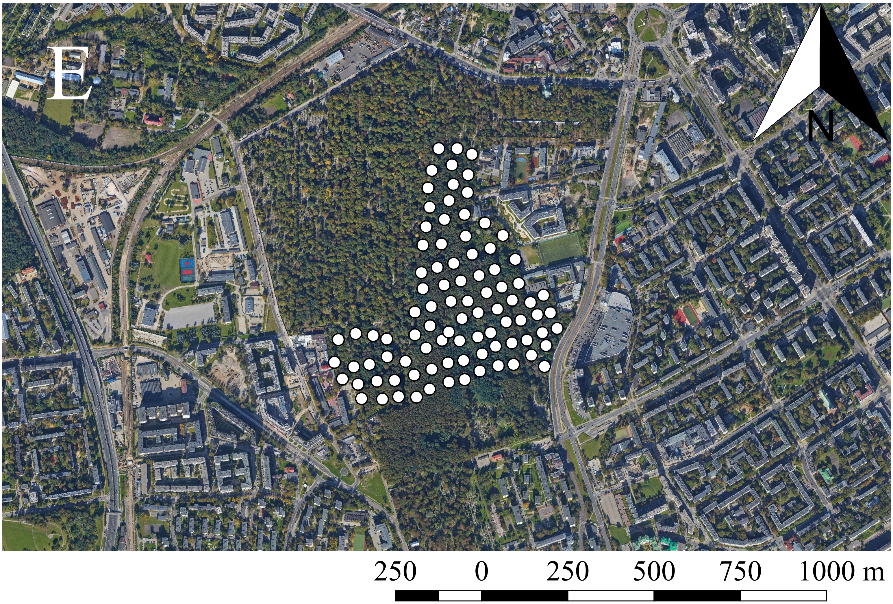

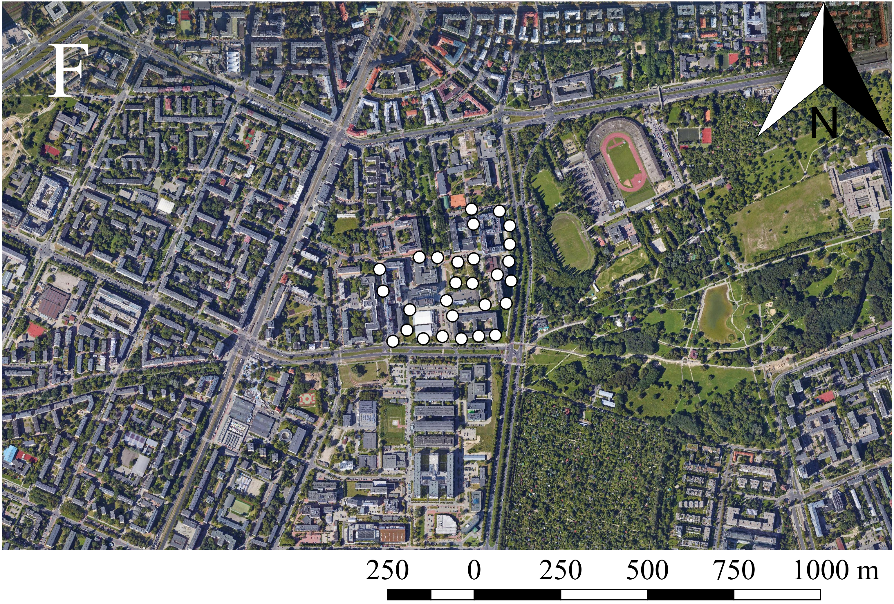

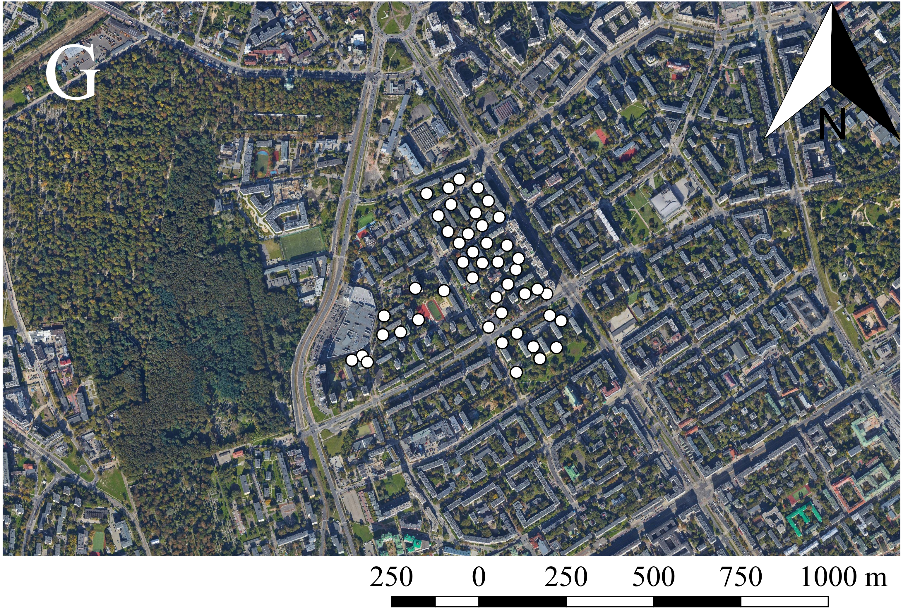

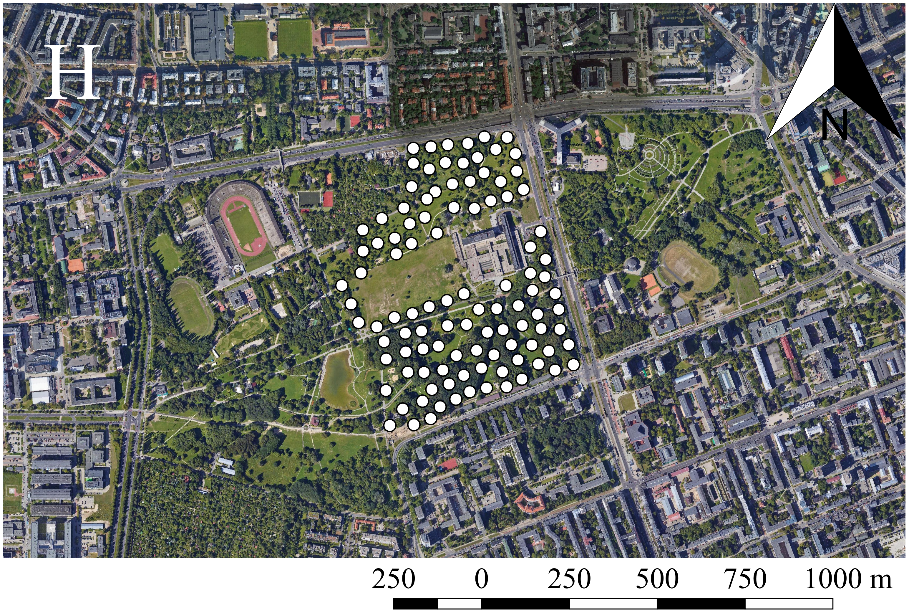


**Figure S1.** Satellite imagery of each study site generated in QGIS (v.3.10.4). Basic maps were obtained using the plugin “*QuickMapServices”* and derived from Google Satellite. Study sites include: a Suburban village (A), a Natural forest (B), two Residential areas (C and G), two Urban woodlands (D and E), an Office area (F) and an Urban park (H). Sites are ordered in decreasing distance to Warsaw city centre. Each white dot indicates a nestbox location.

**Table S1.** **Best fitting model assessed at the population level – (monotonic growth in great tit and blue tit nestlings *– model 12*).**

|  | | | | | | |
| --- | --- | --- | --- | --- | --- | --- |
| **Population** | **Parameters** | **Individuals fitted/n** | **% fitted** | **RSE** | **df. model** | **df. residual** |
| Great tit | *Asym, Infl, k, m* | 76/88 | 86.4 | 0.53 | 304 | 290 |
| Blue tit | *Asym, Infl, k, m* | 151/165 | 91.5 | 0.38 | 604 | 583 |

**Table S1.** Best fitting growth curve parameters and statistics from the R-package *FlexParamCurve* (Oswald et al., 2012). Only dense temporal mass measurements collected in 2016 were used in the analyses, which were run for each species separately. Statistics are detailed in *Materials and* *Methods 2.6.1*.

**Figure S2. Pearson correlation tests of laying date and temperature.**


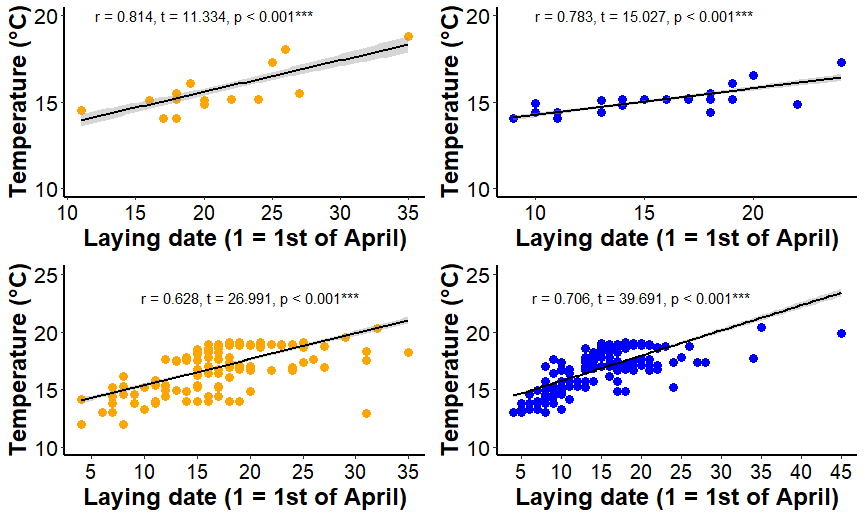


**Figure S2.** Pearson correlation tests of laying date and average temperature. Correlation tests were performed in great tits (yellow dots) and blue tits (blue dots) separately. Analyses were performed on the growth curve parameters dataset (top) which includes only one year of data (2016; n= 67 great tit nestlings and n=144 blue tit nestlings), and on the full dataset (bottom) which includes three years of data (2016-2018; n= 1118 great tit nestlings and n= 1582 blue tit nestlings).

**Table S2. Subsets of LMMs (ΔAIC_c_ < 2) with Gaussian distribution partitioning in great tit and blue tit growth curve parameters.**

| **Species** | **Response** | **Model subset** | **AIC_c_** | **ΔAIC_C_** | **AIC_C_ weight** |
| --- | --- | --- | --- | --- | --- |
|  |  |  |  |  |  |
| Great tit | Asymptotic mass (g) | = Intercept + Sex | 321.9 | 0.0 | 0.659 |
| (n nestlings = 67) |  | = Intercept + Sex + N nestlings | 323.2 | 1.31 | 0.341 |
| (n broods = 15) |  |  |  |  |  |
|  | Inflection point (days) | = Intercept | 205.5 | 0.0 | 1 |
|  |  |  |  |  |  |
|  | Growth rate (k) | = Intercept | -44.2 | 0 | 1 |
|  |  |  |  |  |  |
| Blue tit | Asymptotic mass (g) | = Intercept + Sex | 578.9 | 0.0 | 0.618 |
| (n nestlings = 144) |  | = Intercept + Sex + N nestlings | 579.9 | 0.96 | 0.382 |
| (n broods = 26) |  |  |  |  |  |
|  | Inflection point (days) | = Intercept | 436.8 | 0.00 | 0.695 |
|  |  | = Intercept + ISA | 438.4 | 1.65 | 0.305 |
|  |  |  |  |  |  |
|  | Growth rate (k) | = Intercept | 255.6 | 0.0 | 0.660 |
|  |  | = Intercept + Sex | 257.0 | 1.33 | 0.340 |

**Table S2.** Subset of Linear Mixed Models with Gaussian distribution partitioning in great tit and blue tit growth curve parameters (ΔAIC_c_ < 2).

Data on growth curve parameters refers to dense temporal mass measurements collected in 2016. Great tits and blue tits were analysed separately. Each growth curve parameter (Asymptotic mass, Inflection point and Growth rate) was fitted as response variable. The following variables were included as fixed effects: ISA (percentage of impervious surface area around each nestbox), sex, number of nestlings (“N nestlings” as number of nestlings that hatched in the brood). Brood ID was fitted as random factor within each model.

**Table S3. Subsets of LMMs (ΔAIC_c_ < 2) with Gaussian distribution** **partitioning in great tit and blue tit nestling body mass at consecutive developmental stages.**

| **Species** | **Response** | **Model** | **AIC_C_** | **ΔAIC_C_** | **AIC_C_ weight** |
| --- | --- | --- | --- | --- | --- |
| Great tit | Mass day 2  (n = 869) | = Intercept | 1003.2 | 0.0 | 1 |
|  | Mass day 5  (n = 928) | = Intercept + ISA + Year | 2934.1 | 0.0 | 1 |
|  | Mass day 10  (n = 732) | = Intercept + ISA + Year | 2934.0 | 0.0 | 1 |
|  | Mass day 15  (n = 665) | = Intercept + N nestlings | 2595.8 | 0.0 | 1 |
| Blue tit | Mass day 2  (n = 1144) | = Intercept | 711.2 | 0.0 | 1 |
|  | Mass day 5  (n = 1295) | = Intercept + Year | 3350.6 | 0.0 | 1 |
|  | Mass day 10  (n = 924) | = Intercept + ISA + Laying date + Year | 3046.9 | 0.0 | 1 |
|  | Mass day 15  (n = 839) | = Intercept + ISA + Year | 2646.3 | 0.0 | 1 |

**Table S3.** Subsets of Linear Mixed Effects Models (LMMs; ΔAIC_c_ < 2) with Gaussian distribution partitioning in great tit and blue tit nestling body mass at consecutive stages of development. Data on body mass included measurements obtained on days 2, 5, 10 and 15 after hatching. Body mass measurements were collected in 2016, 2017 and 2018. Body mass was fitted as response variable while the following variables were included as fixed effects: ISA (percentage of impervious surface area around each nestbox), number of nestlings (“N nestlings” as found in the nest at the day of the measurement), laying date (1 = 1^st^ of April), year (2016, 2017, 2018). Brood ID was fitted as random factor.

**Table S4. Subsets of GLMMs (ΔAIC_c_ < 2) explaining variation in nestling survival in great tits and blue tits.**

| **Species** | **Response** | **Model subset** | **AIC_C_** | **ΔAIC_C_** | **AIC_C_ weight** |
| --- | --- | --- | --- | --- | --- |
| Great tit | Early age survival | = Intercept + Laying date | 330.7 | 0.0 | 0.41 |
|  | (n = 1104)  (0 = 115; 1 = 989) | = Intercept | 331.7 | 1.0 | 0.25 |
|  |  | = Intercept + Laying date + NNestlings | 332.1 | 1.4 | 0.19 |
|  |  | = Intercept + Laying date + ISA | 332.6 | 1.9 | 0.15 |
|  |  |  |  |  |  |
|  | Medium age survival | = Intercept + ISA | 502.8 | 0.0 | 0.36 |
|  | (n = 988)  (0 = 133; 1 = 855) | = Intercept + ISA + Year | 502.8 | 0.0 | 0.35 |
|  |  | = Intercept + ISA + Year + Laying date | 504.4 | 1.6 | 0.15 |
|  |  | = Intercept + ISA + Year + N nestlings | 504.7 | 1.9 | 0.14 |
|  |  |  |  |  |  |
|  | Late age survival | = Intercept + ISA + Laying date | 553.1 | 0.0 | 0.46 |
|  | (n = 855)  (0 = 180; 1 = 675) | = Intercept + ISA | 553.6 | 0.5 | 0.35 |
|  |  | = Intercept + ISA + Laying date + N nestlings | 554.9 | 1.8 | 0.19 |
|  |  |  |  |  |  |
|  | Fledging survival | = Intercept | 170.6 | 0.0 | 0.42 |
|  | (n = 665) | = Intercept + N nestlings | 171.6 | 1.0 | 0.26 |
|  | (0 = 29; 1 = 636) | = Intercept + Laying date | 172.5 | 1.9 | 0.16 |
|  |  | = Intercept + ISA | 172.6 | 2.0 | 0.16 |
|  |  |  |  |  |  |
| Blue tit | Early age survival | = Intercept + Year + N nestlings + ISA | 602.7 | 0.0 | 0.54 |
|  | (n = 1568;  (0 = 110;1 = 1458) | = Intercept + Year + N nestlings | 603.0 | 0.3 | 0.46 |
|  |  |  |  |  |  |
|  | Medium age survival | = Intercept + ISA + N nestlings + Year + Laying date | 942.6 | 0.0 | 0.64 |
|  | (n = 1456)  (0=261;1 = 1195) | = Intercept + ISA + N nestlings + Year | 943.7 | 1.1 | 0.36 |
|  |  |  |  |  |  |
|  | Late age survival | = Intercept + ISA + Laying date | 926.3 | 0.0 | 0.71 |
|  | (n = 1195)  (0 = 338; 1 = 857) | = Intercept + ISA + Laying date + N nestlings | 928.1 | 1.8 | 0.29 |
|  | Fledging survival | = Intercept + Year | 426.1 | 0.0 | 0.26 |
|  | (n = 838) | = Intercept + Year + N nestlings | 426.1 | 0.0 | 0.26 |
|  | (0 = 91; 1 = 747) | = Intercept + Year + ISA | 427.3 | 1.2 | 0.14 |
|  |  | = Intercept + Year + N nestlings + ISA | 427.6 | 1.5 | 0.12 |
|  |  | = Intercept + Year + Laying date | 427.7 | 1.6 | 0.12 |
|  |  | = Intercept + Year + N nestlings + Laying date | 428.0 | 1.9 | 0.10 |

**Table S4.** Subsets of Generalized Linear Mixed Models (GLMMs; ΔAIC_c_ < 2) with binomial distribution partitioning in great tit and blue tit nestling survival at consecutive stages of development. Species were analysed separately. Data were collected for three years (in 2016, 2017 and 2018) and included four ages of development: early (whether a nestling survived from 2 to 5 days after hatching), medium (from 5 to 10 days after hatching), late (from 10 to 15 days after hatching) and fledging (from 15 to 25 days after hatching). Survival (binomial variable coded as 0 or 1) was fitted as response variable. Each model included the following fixed effects: ISA (percentage of impervious surface area around each nestbox), number of nestlings (“N nestlings”, as found in the nest at the start of each respective age category), year (2016, 2017 and 2018), laying date (1 = 1^st^ of April). Brood ID was fitted in each model as random effect.

**Table S5. Intensity of selection (selection differentials) on body mass 2 days after hatching in great tits and blue tits.**

| **Species** | **Response** | **Variable** | **Estimate** | **s.e.** | **Z statistic** | **p** |
| --- | --- | --- | --- | --- | --- | --- |
|  |  |  |  |  |  |  |
| **Great tit**  *low ISA* | Fledging survival | Intercept | 9.205 | 1.425 | 6.461 | <0.001*** |
|  | (n = 445) | Mass D2 | 0.398 | 0.394 | 1.011 | 0.312 |
|  |  | N nestlings | 0.461 | 0.792 | 0.582 | 0.561 |
|  |  | Laying date | 0.014 | 0.984 | 0.014 | 0.988 |
| *high ISA* | Fledging survival | Intercept | 12.722 | 1.956 | 6.504 | <0.001*** |
|  | (n = 424) | **Mass D2** | **2.317** | **0.664** | **3.490** | **<0.001***** |
|  |  | N nestlings | 0.116 | 1.472 | 0.079 | 0.937 |
|  |  | Laying date | 0.600 | 1.300 | 0.461 | 0.645 |
|  |  |  |  |  |  |  |
| **Blue tit**  *low ISA* | Fledging survival | Intercept | 8.767 | 1.443 | 6.075 | <0.001*** |
|  | (n = 558) | **Mass D2** | **0.991** | **0.271** | **3.657** | **<0.001***** |
|  |  | N nestlings | 0.551 | 1.139 | 0.484 | 0.628 |
|  |  | Laying date | 0.047 | 0.695 | 0.067 | 0.946 |
| *high ISA* | Fledging survival | Intercept | 10.591 | 1.452 | 7.292 | <0.001*** |
|  | (n = 586) | **Mass D2** | **1.777** | **0.435** | **4.084** | **<0.001***** |
|  |  | N nestlings | -0.249 | 0.907 | -0.274 | 0.784 |
|  |  | Laying date | 0.126 | 1.155 | 0.109 | 0.913 |

**Table S5.** Standardised univariate selection differentials *s* were calculated in low and high ISA environments separately. Positive selection differentials were recorded for mass at day 2, confirming that mass shortly after hatching significantly impacts the survival of both great tits (in high ISA environment) and blue tits (in high and low ISA environments). Estimates of standardised selection differentials were obtained from GLMMs as detailed in *Materials and Methods 2.6.3*. For each species, Fitness *w* was computed as individual survival at fledging (a binomial trait), standardised by yearly average survival for the population. All response variables were z-standardised at an annual level, and included: Trait z, corresponding to mass at day 2 (recorded as soon as possible after hatching to include the trait’s full phenotypic distribution before selection starts operating on the trait), number of nestlings recorded on day 2 and laying date (1=1^st^ of April). Brood ID was fitted as random effect in the models. Mass at day 2 was selected because it is likely to carry a considerable genetic component, which is also least affected by the environment relative to later records of mass. (n) indicates the number of nestlings. **Significance levels: *p<0.05, **p<0.01, ***p<0.001**.

**Table S6. Interactive effects of ISA on selection differentials.**

| **Species** | **Response** | **Variable** | **Estimate** | **s.e.** | **Z statistic** | **p** |
| --- | --- | --- | --- | --- | --- | --- |
| **Great tit** | Fledging survival | Intercept | 11.662 | 1.515 | 7.697 | <0.001*** |
|  | (n = 869) | **Mass D2** | **2.170** | **0.619** | **3.506** | **<0.001***** |
|  |  | low ISA | -1.434 | 1.621 | -0.885 | 0.376 |
|  |  | N nestlings | 0.296 | 0.725 | 0.408 | 0.683 |
|  |  | Laying date | 0.387 | 0.815 | 0.474 | 0.635 |
|  |  | **Mass D2 : low ISA** | **-1.759** | **0.741** | **-2.374** | **0.018*** |
| **Blue tit** | Fledging survival | Intercept | 10.037 | 1.212 | 8.280 | <0.001*** |
|  | (n = 1144) | **Mass D2** | **1.698** | **0.413** | **4.108** | **<0.001***** |
|  |  | low ISA | -0.451 | 1.313 | -0.343 | 0.732 |
|  |  | N nestlings | -0.074 | 0.575 | -0.129 | 0.898 |
|  |  | Lay date | 0.341 | 0.810 | 0.421 | 0.674 |
|  |  | Mass D2 : low ISA | -0.684 | 0.492 | -1.389 | 0.165 |

**Table S6.** For each species, interactive effects between ISA and mass on Day 2 were tested by merging low and high ISA datasets together and by fitting an interaction between mass at D2 and ISA categories (low vs. high ISA). In all other aspects, model structure was the same as in Table S6. A directional, positive selection differential on body mass was recorded in both species (Table S6), and point estimates of selection differentials were always larger in high ISA environments (Table S6). Importantly, the selection differential in high ISA environment was significantly larger than in low ISA environment for great tits (Table S7), but not for blue tits (Table S7, Figure 3). (n) indicates the number of nestlings. **Significance levels: *p<0.05, **p<0.01, ***p<0.001**.

**Table S7. Linear mixed effect models estimating the effect of ISA on nestling body mass measured at consecutive developmental stages.**

| **Species** | **Age (days)** | **n** | **Mean (± SD)** | **Estimate** | **s.e.** | **t-value** | **p** |
| --- | --- | --- | --- | --- | --- | --- | --- |
| **Great tit** | **2** | **869** | **1.99 (± 0.52)** | **-0.006** | **0.002** | **-2.898** | **0.004**** |
| Blue tit |  | 1144 | 1.45 (± 0.39) | -0.002 | 0.001 | -1.538 | 0.126 |
|  |  |  |  |  |  |  |  |
| **Great tit** | **5** | **928** | **5.37 (± 1.38)** | **-0.019** | **0.005** | **-3.681** | **<0.001***** |
| Blue tit |  | 1295 | 3.57 (± 1.01) | -0.006 | 0.003 | -1.867 | 0.064 |
|  |  |  |  |  |  |  |  |
| Great tit | 10 | **732** | **12.05 (± 2.51)** | **-0.057** | **0.011** | **-4.944** | **<0.001***** |
| Blue tit |  | **924** | **7.72 (± 1.7)** | **-0.026** | **0.008** | **-3.249** | **0.001***** |
|  |  |  |  |  |  |  |  |
| Great tit | 15 | **665** | **15.14 (± 2.3)** | **-0.035** | **0.013** | **-2.692** | **0.008**** |
| Blue tit |  | **839** | **9.68 (± 1.62)** | **-0.026** | **0.008** | **-3.205** | **0.001**** |

**Table S7**. Linear mixed effects models (LMMs). ISA was fitted as explanatory variable and Brood ID was fitted as random factor in all models. (n) indicates the number of nestlings. Significance levels: *p<0.05, **p<0.01, ***p<0.001.

**References:**

Oswald, S. A., Nisbet, I. C. T., Chiaradia, A., & Arnold, J. M. (2012). FlexParamCurve: R package for flexible fitting of nonlinear parametric curves: *Nonlinear parametric curve-fitting*. *Methods in Ecology and Evolution*, **3**(6), 1073–1077. https://doi.org/10.1111/j.2041-210X.2012.00231.x
